# Potent hepatoprotective activity of common rattan (*Calamus rotang* L.) leaf extract and its molecular mechanism

**DOI:** 10.1186/s12906-023-03853-9

**Published:** 2023-01-30

**Authors:** Walaa S. Anwar, Fatma M. Abdel-maksoud, Ahmed M. Sayed, Iman A. M. Abdel-Rahman, Makboul A. Makboul, Ahmed M. Zaher

**Affiliations:** 1 Department of Pharmacognosy, Faculty of Pharmacy, Merit University, New Sohag, Egypt; 2 Department of Anatomy and Embryology, Faculty of Vet. Medicine, Assiut University, Assiut, Egypt; 3 Biochemistry Laboratory, Chemistry Department, Faculty of Science, Assiut University, Assiut, Egypt; 4 Department of Pharmacognosy, Faculty of Pharmacy, South Valley University, Qena, Egypt

**Keywords:** *Calamus rotang*, Hepatoprotective, Molecular docking, Apoptosis, HPLC, Metabolite profiling

## Abstract

**Background:**

*Calamus rotang* L. (CR) is an Indian shrub. The leaves and other organs of the plant are traditionally used in India for treatment of various diseases. The in vitro antioxidant property of the leaves extract was previously established. Thus, the current study aimed to evaluate the antioxidant and hepatoprotective effects of CR ethyl acetate extract at a dose of 350 mg/kg on CCl_4_ induced hepatotoxic rats through different mechanisms.

**Methods:**

Histopathological examination of the treated rats’ group in comparison with positive and negative controls were performed. Quantitative measuring of the proinflammatory cytokines (TNF α), inflammatory regulators (Arginase, PPAR α) and the antiapoptotic protein Bcl-2 in comparison with positive and negative control groups was achieved using immunohistochemical examination. HPLC profiling of the polyphenol contents and molecular docking of the identified compounds against BH3 proapoptotic protein were correspondingly studied to evaluate the potential antiapoptotic property.

**Results:**

The CR extract greatly protects the liver tissue through the suppression of TNF α, arginase and PPAR α induced by CCl_4_ as well as its enhancement of the antiapoptotic Bcl-2 protein. Fourteen polyphenols of different classes were identified in CR extract and tested via molecular docking for their potential antiapoptotic activities against BH3 protein. Naringin, rutin, 7-hydroxy flavone, and ellagic acid compounds exhibit the highest affinity and potential inhibition of pro-apoptotic protein BH3 via molecular docking study.

**Conclusions:**

The ethyl acetate fraction of the leaves of *C. rotang* is rich in polyphenols that exhibited potent hepatoprotective effect on CCl_4_ induced hepatotoxic rats through its antioxidant, anti-inflammatory, anti-steatosis and antiapoptotic properties.

**Supplementary Information:**

The online version contains supplementary material available at 10.1186/s12906-023-03853-9.

## Introduction

The liver is a site of metabolism and detoxification of chemicals, drugs, and harmful environmental toxicants. It protects the whole-body organs from injury by those harmful toxins. The exposure to liver toxins in food, water, air and medicines for a long time causes acute liver injury, cirrhosis, fibrosis, fatty liver and cancer. These diseases are worldwide spread nowadays. In some critical cases, urgent liver transplantation is required to save the life of patients [1].

Halogenated alkanes such as carbon tetrachloride (CCl_4_), chloroform and iodoform are examples of chemical toxins that have been restricted due to their high risk of hepatotoxicity [2, 3]. Liver enzymes activate the halogenated alkanes (CCl_4_) into highly reactive free radicals such as trichloromethyl and trichloromethyl peroxyl radicals (**·**CCl_3_ and **·**CCl_3_O_2_). These radicals interact with hepatic cell proteins, fats and nucleic acids which harm the normal cellular processes such as lipid metabolism, hepatic enzyme reactions, initiation of cancer, liver fibrosis, and finally cell death [2]. Therefore, antioxidants such as plant polyphenols (flavonoids and tannins) have an important role in scavenging the free radicals (**·**CCl_3_ and **·**CCl_3_O_2_) [4] and protect the hepatic cells from their liver toxicity [5, 6].

Kupffer cells are the main players in liver inflammation. They are localized macrophages in the liver and are responsible for the expression and release of cytokines upon their activation during liver injury. They exhibit markers such as M1-like macrophages or M2-like macrophages that regulate liver inflammation [7]. Several inflammatory cytokines such as chemokines, IL-6, IL-12, IL-18, and TNFα are released from Kupffer cells in response to liver injury, alcoholic liver disease, and other hepatic toxins [7]. Other bone-marrow derived macrophages, such as M1 and M2 macrophages, play a role in liver inflammation by releasing inflammatory cytokines (TNF-α, IL-1, and IL-6) [7]. After liver inflammation, hepatic macrophages such as Kupffer cells and macrophage-TGF-β1 activate myofibroblasts (hepatic stellate cells) to produce fibrosis [7].

CCl_4_, a liver toxin, induces Kupffer cells to release several inflammatory mediators such as TNFα, TGF-ß, and IL-1, nitric oxide, IL-10 and IL-6 which are responsible for necrosis and fibrosis of hepatic cells [8]. Polyphenols (flavonoids and tannins) are promising anti-inflammatory compounds that inhibit the release of the inflammatory mediators by several mechanisms and hence, protect the hepatic cells from necrosis and fibrosis [9].

Several plant extracts exhibited potent in vivo hepatoprotective activity against CCl_4_ induced hepatotoxicity. For example, the methanol and chloroform extracts as well as some isolated phenolics from *Aframomum melegueta* seeds exhibited hepatoprotective activity on CCl_4_ induced hepatotoxic rats. Suppression of inflammatory response of cytokines, apoptosis and enhancement of the antioxidant defense activity were the mode of action [10]. *Lannea stuhlmannii* and *Lannea humilis* tannins rich extracts displayed a potent hepatoprotective effect through the enhancement of the anti-apoptotic protein Bcl-2 [11]. Strawberry (*Fragaria ananassa*) juice showed enhancement of the anti-apoptotic Bcl-2 protein and controlled the pro-apoptotic Bax and caspase-3 proteins with a clear reducing collagen areas in hepatic tissue [12]. Phenolic extract of barley (*Hordeum vulgare*) exhibited a hepatoprotective effect on hepatotoxic mice induced by CCl_4_ [13]. The elevation of liver antioxidant enzymes and reducing the hepatocyte apoptosis were the mechanism of action of the phenolic extract of barley [13].

*Calamus rotang* L. (Rattan palm) a monocot climbing shrub, belongs to family Ericaceae. It is native to South-West Asia. In India, the shrub is cultivated for its’ edible fruits and medicinal importance especially in Ayurveda. The shoots are used as anthelmintic and the leaves are used for eye problems, skin diseases, pruritus and cough [14–16].

Few biological studies on *C. rotang* (CR) were previously reported. For example, the aqueous extract of leaves showed immunoadjuvant activity against hepatitis B surface antigen [17]. The in vitro antioxidant study on fruits and leaves methanol extracts revealed that the leaves have more polyphenolics than the fruits [18]. The methanol extract of seeds displayed in vivo antidiabetic and hypoglycemic activities [19]. The phytochemical study on *C. rotang* rhizome led to isolation of ( +) –afzelechin, *β*-sitosterol and *β*-sitosterol glucoside [20]. No chemical study was previously reported on the leaves, fruits and seeds. The current study aimed to determine the total phenolics, total flavonoids, antioxidant property and in vivo hepatoprotective activity of the CR extract on CCl_4_ induced hepatotoxic rats. HPLC chemical characterization of the polyphenols (flavonoids and phenolics) in CR extract was performed. The binding ability of the identified compounds to the pro-apoptotic Bcl-2: Bim (BH3) protein by molecular docking was studied.

## Material and methods

### Solvents and chemicals

The solvents used in this study for extraction (methanol) and chromatography (methanol and acetonitrile) were HPLC solvents (Thermo Fisher Scientific, Pittsburgh, USA). The chemicals, such as Folin-Ciocalteu’s phenol reagent, gallic acid, quercetin, AlCl_3_, DPPH, and ascorbic acid, used in the determination of phenolic and flavonoid contents and antioxidant activity were purchased from Sigma (Sigma-Aldrich, Burlington, MA, USA). Hematoxylin and eosin kits and toluidine blue dye used in the histopathological study were bought from Abcam (Cambridge, UK). The following antibodies: anti-TNFα antibody (sc-52746), anti-PPARα antibody (sc-398394), anti-arginase (sc-271430), and anti-Bcl-2 antibody (sc-7382) used in the immunohistochemistry study were purchased from Santa Cruz Biotechnology (Texas, USA).

### Plant materials

The leaves of *C. rotang* L. were used in the current study. They were collected in October 2018 from Aswan Botanical Garden, Aswan, Egypt. The plant was kindly identified by Dr. Amr M. M. Mahmoud, Director of Aswan Botanical Garden, Hort. Res. Institute, Agriculture centre, Egypt. Voucher specimen of the plant leaves was placed in the herbarium of the Pharmacognosy Department, Faculty of Pharmacy, Assiut University, Egypt (Voucher no. A20220906).

### Extraction and fractionation

A hundred grams of dried powdered leaves were macerated in methanol (500 mL) at room temperature (25 °C) with shaking for 24 h. The extraction process was repeated twice until exhaustion. The methanol extract was combined and concentrated under reduced pressure using rotavapor. The fractionation of the dried methanol extract (10 g) was performed by suspending it in 250 mL of 10% methanol in water using a 1L separating funnel. Liquid–liquid extraction to fractionate the aqueous extract using dichloromethane and ethyl acetate solvents (250 mL × 3 for each solvent) was performed. The dichloromethane and ethyl acetate fractions were collected and concentrated at reduced pressure to yield dichloromethane extract (3 g), ethyl acetate extract (2 g), and aqueous extract (5 g). The preliminary screening test for flavonoids demonstrated that the ethyl acetate fraction is the richest flavonoid fraction.

### Total phenolic contents

The total phenolic content of CR extract was determined according to the method previously reported [21]. Briefly, gallic acid was used as a standard phenolic compound. Serial dilution of 100 ppm gallic acid solution was prepared (100–10 ppm) in distilled water. Folin-Ciocalteu’s phenol reagent (0.2 mL) was mixed with all gallic acid solutions and tested CR ethyl acetate extract (1:1). One mL of sodium carbonate and 1.6 mL distilled water were added to the mixtures after 5 min. All tested and standard mixtures were kept on dark for 30 min. After that, the samples were centrifuged, and their colorimetric absorbance was measured in triplicates at 750 nm using UV2000 spectrophotometer (Ray Wild Limited company, L569 Gottingen, Germany). Standard curve of gallic acid was obtained (Fig. S3) and the total phenolic content of CR extract GAE mg/g dry weight was calculated as a mean of triplicate measurements ± SD.

### Total flavonoid contents

The total flavonoid content of CR extract was determined according to the method previously reported [22]. Serial dilution of 100 ppm quercetin solution was prepared (100–10 ppm) in methanol. AlCl_3_ 2% reagent (0.6 mL) was mixed with all quercetin solutions and tested CR ethyl acetate extract (1:1). All tested and standard mixtures were kept for 60 min at room temperature. After that, the samples were measured for their colorimetric absorbance in triplicates at 415 nm using UV2000 spectrophotometer (Ray Wild Limited company, L569 Gottingen, Germany) Standard curve of quercetin was obtained (Fig. S4) and the total flavonoid content of CR extract QE mg/g dry weight was calculated as a mean of triplicate measurements ± SD.

### Antioxidant DPPH assay

The capacity of CR extract to scavenge free radicals was calculated by DPPH previously reported method [23]. DPPH stock solution of 200 µM, tested extract samples (60—1000 µg/mL ethanol) and ascorbic acid reference samples (60—1000 µg/mL ethanol) were prepared. The antioxidant reaction was performed by adding 300 µL DPPH solution to 300 µL of each tested or control sample on dark for 30 min at room temperature. The colorimetric absorbance was measured at 517 nm. The percentage antioxidant activity was calculated by the following equation SA% = (A_0_—A_s_/A_0_) × 100 (A_0_ = Absorbance of DPPH solution in ethanol, A_s_ = Absorbance of CR extract and DPPH).

### HPLC analysis of CR ethyl acetate extract

#### Detection of phenolic compounds

Twenty-five microliter of CR was injected in HPLC-Agilent 1100 with UV/Vis detector (Agilent technology, USA). The phenolic compounds were separated on C18 column (125 mm × 4.60 mm, 5 µm) using gradient solvent elution. Two solvents were used as mobile phase for separation of phenolics, solvent A: acetic acid in water (1:25) and solvent B: 100% methanol. The gradient solvent method was started with 100% of solvent A for 3 min, followed by 50% solvent B for 5 min, 80% solvent B for next 2 min and finally 50% solvent B for 5 min. Phenolic compounds were detected at 280 nm. They were detected using Agilent ChemStation software (Agilent technology, USA) and were identified by using authentic samples.

#### Detection of flavonoids compounds

Twenty-five microliter of CR was injected in HPLC-Agilent 1100 with UV/Vis detector (Agilent technology, USA). The phenolic compounds were separated on C18 column (250 mm × 4.60 mm, 5 µm) using isocratic solvent elution in 15 min. Two solvents were used as mobile phase for separation of flavonoids, solvent A: formic acid in water (1%) and solvent B: 100% acetonitrile. Seventy % of solvent B was used as isocratic eluted mobile solvent of the experiment. The detector wavelength was 320 nm. The identification of flavonoids was performed by using authentic samples.

#### Animals and experimental design

Eighteen Wistar albino rats weighting 180 to 250 g (aged 6 weeks) were obtained from Animal Housing Center, Faculty of Medicine, Assiut University. They were housed, provided feed and water ad libitum under standard conditions for 7 days before experiments begun. This experiment was carried out in accordance with relevant guidelines and regulations. It was approved by the Institutional Animal Care and Use Committee of the Faculty of Pharmacy, Assiut University (Approved No. S27-22). The rats were divided into three groups; control, CCl_4_ intoxicated and CR extract treated groups. CCl_4_ was chosen to intoxicate the hepatic cells in the positive and treated groups based on a previously published protocol of CCl_4_ induced hepatotoxicity in rats [24–27]. The rats of treated (CR extract) group were supplied orally by 0.3 mL of 350 mg/kg CR extract once a daily for 21 days [28]. The dose of ethyl acetate extract was determined in the range of the previous reported doses of hepatoprotective medicinal plants rich in flavonoids and phenolics [29, 30]. The rats of other groups were supplied orally by 0.3 mL of saline once a daily for 21 days. The rats of all groups except the control were supplied orally with 0.25 mL of 1% CCl_4_ in olive oil per day from day 10. The rats were fasted for 4 h and the animals killed at the end of the corresponding experimental periods. The animals were anesthetized by pentobarbital (50 mg/kg, i.p.). After the rat loss the reflexes, the rat’s thoracic cages had been incised and transcardially perfused with normal saline followed by 4% paraformaldehyde fixative. Liver specimens were obtained after whole-body perfusion of experimental rats with 4% paraformaldehyde. All methods are reported in accordance with ARRIVE guidelines (https://arriveguidelines.org).

#### Histopathological examination

The dissected samples were processed and stained with hematoxylin & eosin and Crossmon’s trichrome (Fig. S5) according to the standard protocols [31]. Histopathological studies were performed using an Olympus CX 41RF light microscope (Olympus Corporation, Tokyo, Japan). Also, specimens from liver were used for semithin sections and stained with toluidine blue [32].

#### Immunohistochemical examination

Immunohistochemistry staining was performed according to the previous reported strudy [33]. The sections were incubated overnight at 4 °C with the following antibodies: anti-TNFα antibody (sc-52746), anti-PPARα antibody (sc-398394), anti-arginase (sc-271430) and anti-Bcl-2 antibody (sc-7382). Immunohistochemical staining was evaluated by Olympus CX 41RF light microscope (Olympus Corporation, Tokyo, Japan).

#### Quantitative analysis of TNFα, Arginase I, BCL2, PPARα immunostaining

The morphometric studies carried out on the immunohistochemical images of the liver of all groups using Image-J software. The measurements were done according to the previous study [34].

### Statistical analysis

The data of immunohistochemical analysis were summarized in figures using “GraphPad Software” (Version 6.05, International Scientific Community) to compare between different variables in CCl_4_, CR extract treated animals in relation to control group. Differences were considered significant if *P* < 0.05 (*). All data were statistically analyzed using Tukey’s test.

### Molecular docking

Molecular docking was conducted by using Autodock [35] vina 1.5.6 software to assess the binding affinities of the selected polyphenols with antiapoptotic protein Bcl-2: (BH3). The crystal structure of BH3 with its inhibitor with PDB ID: 4B4S was retrieved from Protein Data Bank [36]. The interaction residues of the binding site of BH3 that was used for the docking study were identified based on the previous literature [11, 36]. The ligand and all the water molecules were removed from the crystal structure. The polar hydrogen atoms were added and pdbqt file was generated by using AutoDock tools-1.5.6 [37]. The site of the grid box was set at -8.425, 9.62, and -2.654 Å (for × , y and z) by means of a grid of 40, 40, and 40 points (for × , y and z). The binding affinities of polyphenols with Bcl-2 were predicted based on the average of the lowest energy of docking. Chimera [38] 1.12 software was used to analyze and visualize the best-scored conformation.

## Results

### Determination of total phenolics, total flavonoids and antioxidant properties of *C. rotang* L. ethyl acetate

The total phenolics content of *C. rotang* ethyl acetate extract was determined by using gallic acid as a reference compound in Folin-Ciocalteu assay [21]. It contains 804 ± 0.18 mg Gallic Acid Equivalent (GAE)/100 g dry weight as shown in Fig. S1A. The quantity of flavonoids in *C. rotang* ethyl acetate fraction (Fig. S1A) was determined as 1760 ± 0.69 mg Quercetin Equivalent (QE)/100 g dry weight using quercetin as a reference compound in Aluminum chloride colorimetric assay [22]. The CR extract displayed potent DPPH scavenging activity with IC_50_ = 0.12 mg/mL using ascorbic acid as a reference compound in the DPPH assay (Fig. S1B.).

### HPLC analysis of *C. rotang* L. leaves ethyl acetate extract

**Fig. 1:**
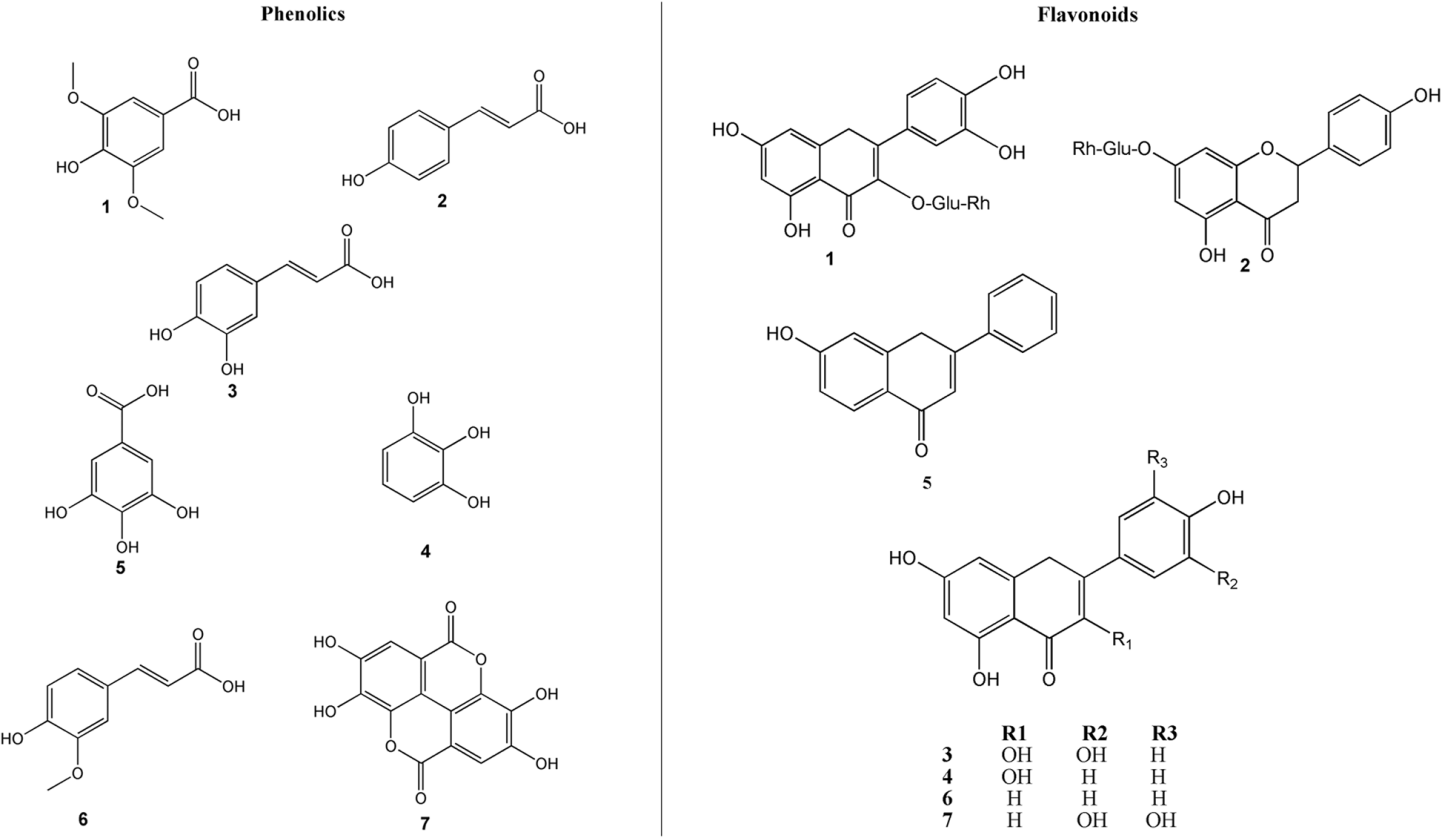
Chemical structures of phenolics and flavonoids detected by HPLC analysis of *C. rotang* L. ethyl acetate extract

CR leaves extract was analyzed by using HPLC–UV for detection of flavonoids and phenolics. Authentic compounds were used to identify the components of CR extract. Fourteen phenolics and flavonoids were detected as shown in HPLC chromatograms (Fig. S2). All the identified compounds are listed in Table 1 and Fig. 1.

**Table 1:** List of compounds detected in *C. rotang* ethyl acetate leaves extract by HPLC

**Peaks no**	**Rt** (Retention time in min)	**Compounds**	**Concentration** **(µg/mL)**
**Phenolics**			
1	5.0	Syringic acid	4.26
2	6.0	*P*-coumaric acid	9.56
3	8.2	Caffeic acid	2.69
4	9.0	Pyrogallol	6.47
5	10	Gallic acid	1.98
6	11	Ferulic acid	0.98
7	13	Ellagic acid	0.46
**Flavonoids**			
1	3.0	Rutin	1.33
2	3.9	Naringin	2.14
3	5.0	Quercetin	6.88
4	6.0	Kaempferol	3.04
5	8.9	7-OH flavone	7.15
6	10.0	Apigenin	5.44
7	11.0	Myricetin	4.63

### Histopathological findings

**Fig. 2:**
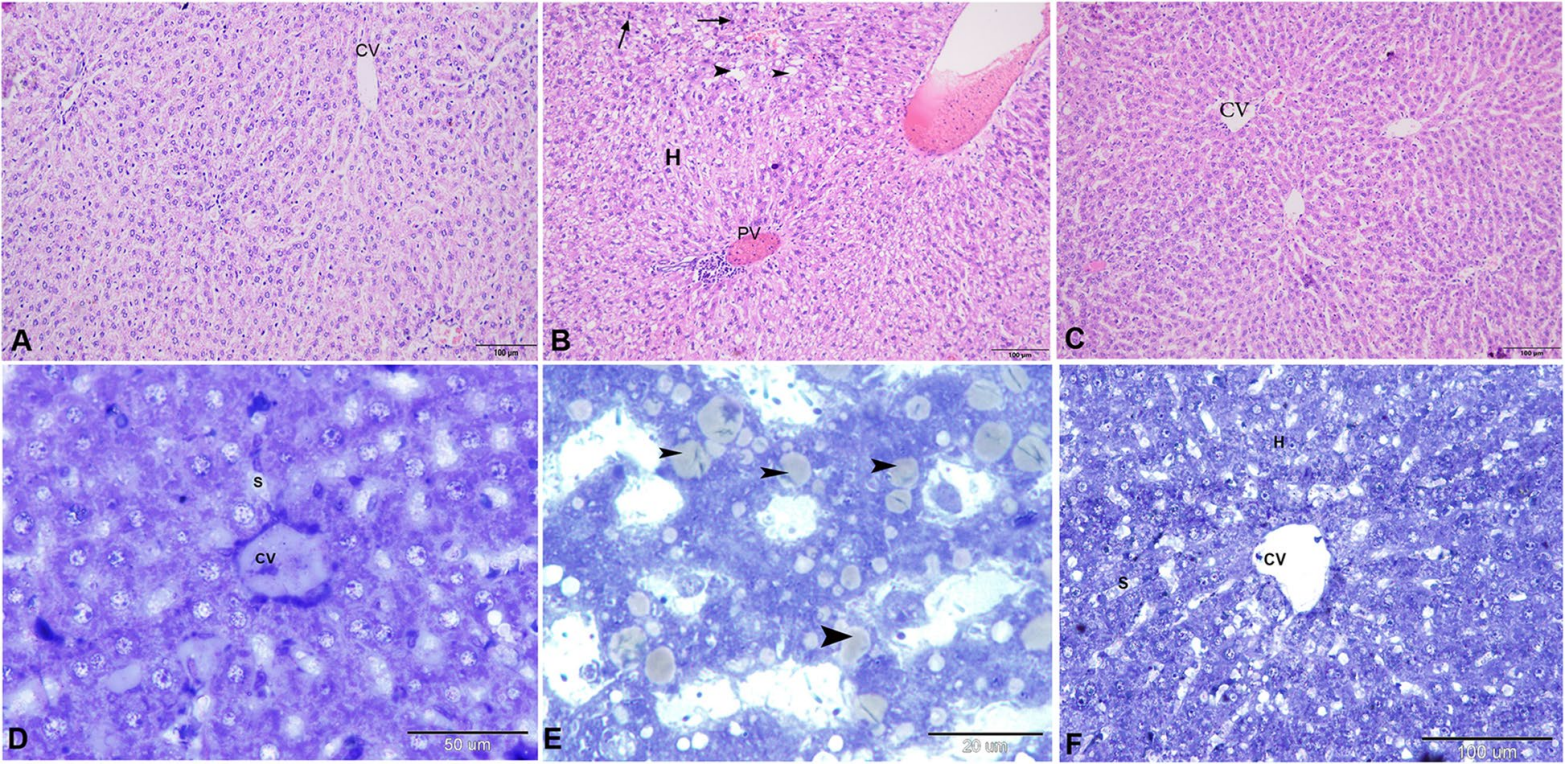
Photomicrograph of a liver sections stained with H & E (**A**-**C**) and semithin sections stained by Toluidine blue (**D**-**F**). **A**, **D** showing the normal architecture of hexagonal liver lobules with apparently intact hepatocyte, central vein (CV), and blood sinusoids (S). **B**, **E** Liver section of rat treated with CCl_4_, showing distortion of the normal hepatocyte’s appearance with vacuolated cytoplasm, congested portal (PV) and central veins, as well as dilated blood sinusoids, mononuclear cellular infiltration (arrow) and steatosis (arrow heads). **C**, **F** Liver section of rat treated with CR extract group, showing the hepatocytes appearance improved with lesser and smaller vacuoles

The liver in the control group exhibited the normal appearance of hepatic lobules with hexagonal hepatocytes, sinusoidal spaces, and central vein (Fig. 2A & D). However, in the CCl_4_ treated group, the normal hepatic architecture was distorted; hepatocytes showed vacuolated cytoplasm, the central and portal veins were dilated and congested with blood (Fig. 2B). Also, there was a mononuclear cellular infiltration and steatosis (Fig. 2B & E). CR ethyl acetate extract group exhibited potent protection of the hepatic tissue from the histopathological changes caused by CCl_4_ (Fig. 2C & F). There was no or minor vacuolation of the hepatocytes, no sinusoidal dilation or the central and portal veins with slight dilation and congestion were recorded (Fig. 2C & F).

### Immunohistochemical findings

**Fig. 3:**
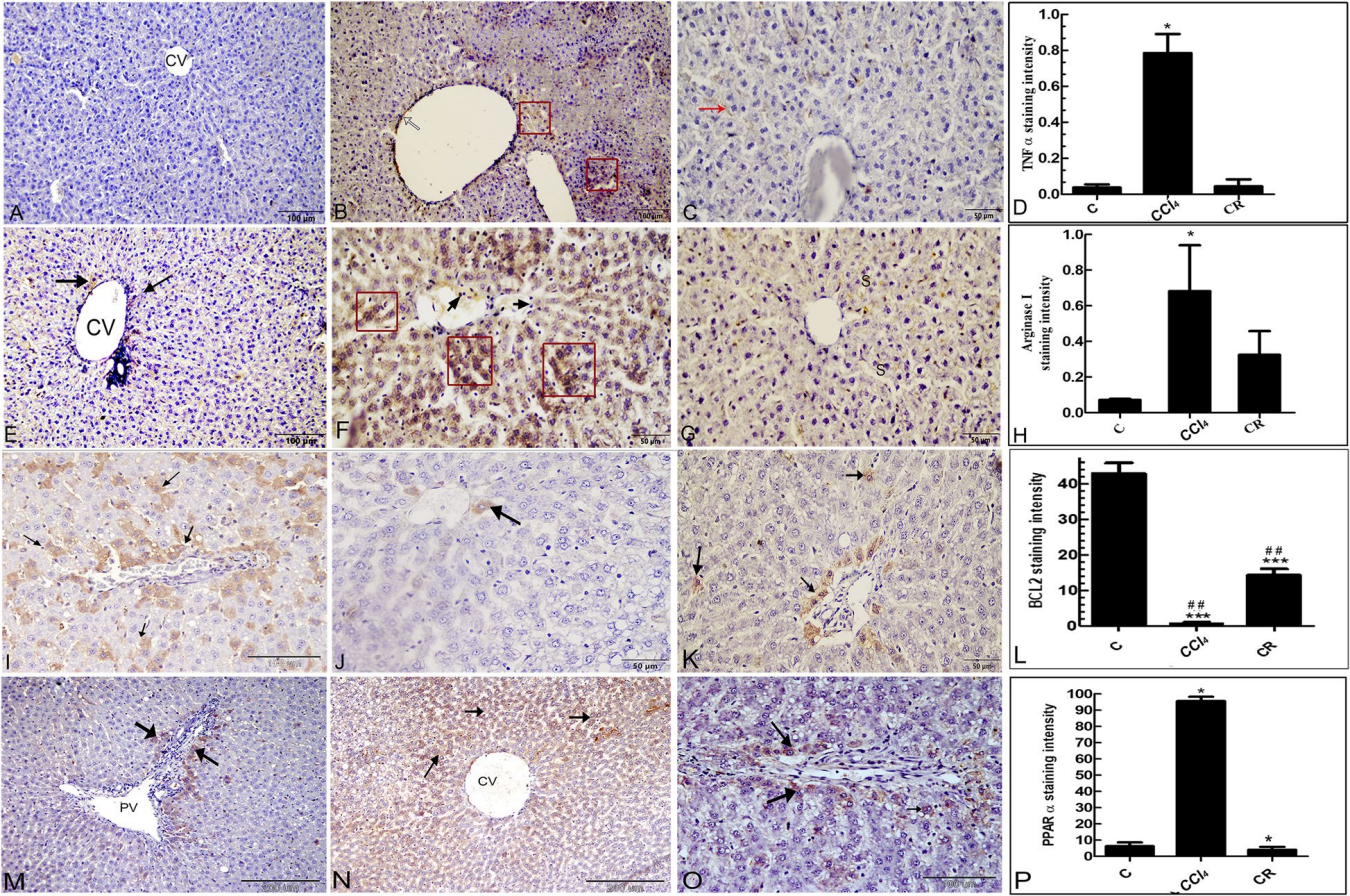
Immunohistochemical staining: **A** Normal hepatocytes showing a weak immunoreactivity against TNF α. **B** CCl_4_ treated groups, showing strongest immunoreactivity in Kupffer cells and endothelium of the central vein. **C** CR treated group, is showing lesser immunoreactivity. **D** Staining quantification of TNF α using image j software. **E** Normal hepatocyte is showing a weak immunoreactivity against Arginase I. **F** CCl_4_ treated group, the cytoplasm of the hepatocytes (red squares) exhibiting strong reactivity within the hepatocyte’s cytoplasm and Kupffer cells (short arrows). G: The hepatocytes show lesser immunoreactivity in CR ethyl acetate extract. **H** Staining quantification of Arginase I, using image j software. **I** Increasing in the number of the Bcl-2 positive hepatocytes (arrows). **J** CCl_4_ treated group, is showing decrease in Bcl-2 positive cells. K: CR ethyl acetate extract treated groups. **L** Staining quantification of Bcl-2 using image j software. **M** Normal group, the cytoplasm of the periportal hepatocytes is showing a staining against PPAR α (arrows). **N** CCl_4_ treated group, is showing an increase in PPAR α positive cells. **O** CR ethyl acetate extract treated group. **P** Staining quantification of PPAR α using image j software. The CR extract treated groups is compared to control and CCl_4_ groups using Tukey test (*n* = 3). *P ≤ 0.05

The Kupffer cells (resident macrophage) and T-cells in the liver produces proinflammatory cytokines like TNF α [39]. TNF α strongly expressed in hepatocytes, endothelium sinusoids and Kupffer cells in CCl_4_ treated group in relative to the normal and CR treated groups (Fig. 3A-D). Arginase is a manganese-containing enzyme, it catalyzes the last stage in the urea cycle, that is responsible for conversion of arginine to urea [40]. Arginase strongly immunoreacted in hepatocytes of the CCl_4_ treated group in relative to the normal and CR treated groups (Fig. 3E-H). Additionally, Bcl-2 is an anti-apoptotic marker, showed a significant increase in hepatocytes immunostaining of the normal and CR treated groups in comparison with CCl_4_ treated group (Fig. 3I-L). The peroxisome proliferator-activated receptors alpha (PPARs α) are transcription factor responsible for the catabolism of the fatty acids in liver [41]. In the CCl_4_ treated group the immunostaining of PPAR α was significantly higher in relation to the normal and CR treated groups (Fig. 3M-P).

### Molecular modelling study

**Fig. 4:**
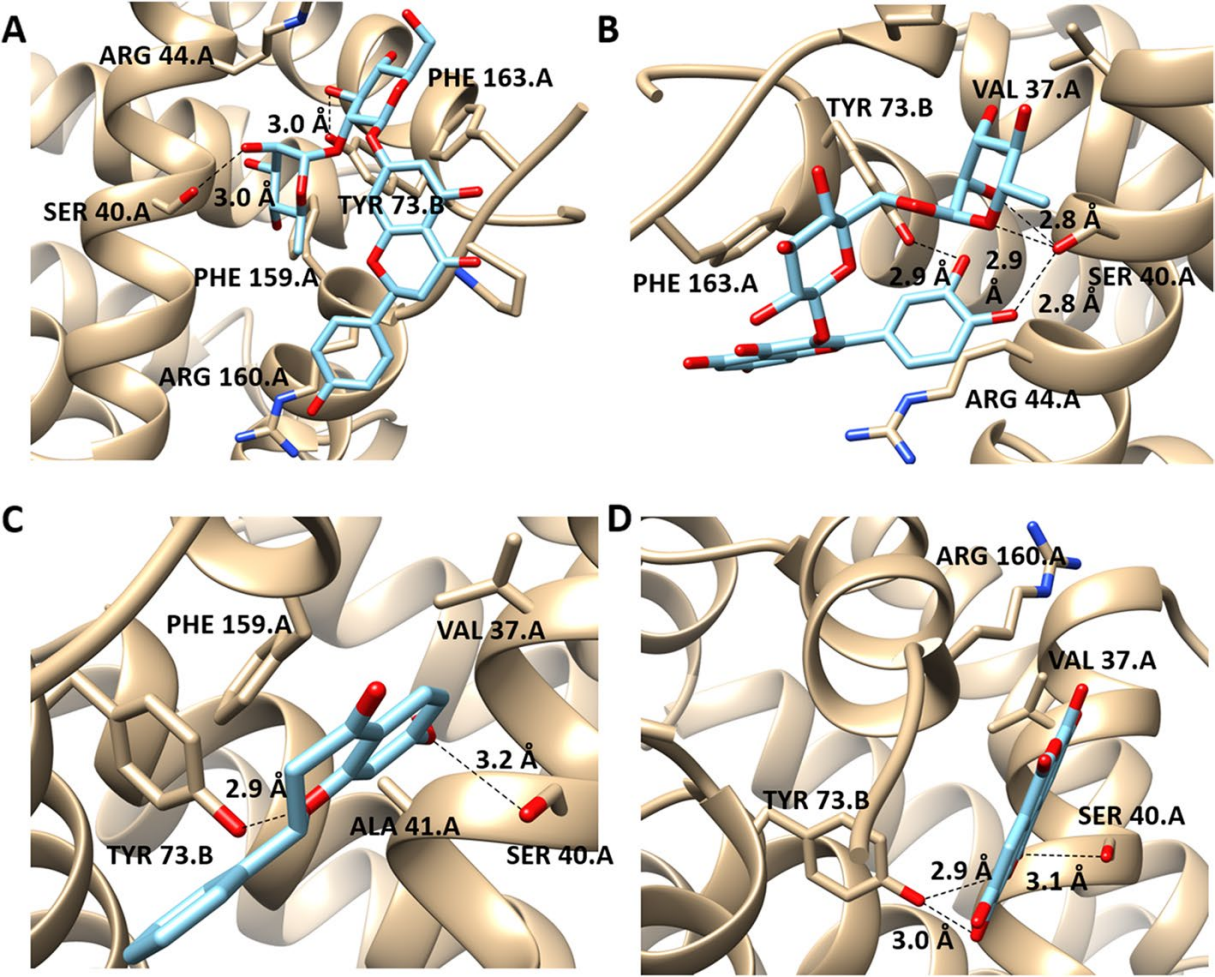
3D-docking poses of Naringin (1A), Rutin (1B), Hydroxy flavone (1C), and Ellagic acid (1D) docked to Bcl-2: Bim (BH3). The binding affinities of Naringin, rutin, 7-hydroxy flavone, and ellagic acid with Bcl-2: Bim (BH3) are -6.88 ± 0.06, -6.70 ± 0.19, -6.42 ± 0.31 and -6.57 ± 0.26 kcal/mol respectively

Naringin, rutin, 7-hydroxy flavone, and ellagic acid exhibited the highest binding affinities with Bcl-2: Bim (BH3) among all the studied polyphenols with docking energy of binding -6.88 ± 0.06, -6.70 ± 0.19, -6.42 ± 0.31 and -6.57 ± 0.26 kcal/mol respectively. All the binding affinities of the polyphenols are listed in Table S1. Naringin formed two hydrogen bonds with the side chains of SER40 and TYR73 at the binding site of Bcl-2: Bim (BH3) (Fig. 4A). Rutin formed a unique hydrogen bonds pattern with the binding site of Bcl-2: Bim (BH3), it formed three hydrogen bonds with SER40 and one more with TYR73. Also, its aromatic ring formed aryl–aryl interactions with the aromatic ring of PHE163 (Fig. 4B). Hydroxy flavone formed two hydrogen bonds with the side chains of SER40 and TYR73 (Fig. 4C). Ellagic acid formed two hydrogen bonds with SER40 and one more hydrogen bond with TYR73 (Fig. 4D).

## Discussion

The CR leaf extract displayed potent DPPH scavenging activity, with IC_50_ = 0.12 mg/mL. The DPPH scavenging of the CR increased with concentration. The higher the concentration of the CR extract, the greater the scavenging of DPPH radicals (Fig. S1).

The most natural plant components that are responsible for antioxidants are flavonoids and phenolics [42, 43]. The first quantitative determination of flavonoids and phenolics in the CR leaf extract was reported in the current study. The total phenolic content of the CR extract was 804 ± 0.18 mg GAE/100 g, and the total flavonoid content was 1760 ± 0.69 mg QE/100 g (Fig. S1). Therefore, CR is a rich source of flavonoids and phenolics in comparison with the previously reported data on antioxidant plants [44–46]. The HPLC characterization of phenolics in CR extract led to the identification of 6 simple phenolic acids; gallic acid, ellagic acid, syringic acid, *p*-coumaric acid, caffeic acid and ferulic acid in addition to one phenolic compound (pyrogallol) as shown in Fig. 1. Additionally, seven flavonoid compounds of different classes; flavonols (Rutin, quercetin, and kaempferol), flavones (Apigenin, 7-OH flavone and myricetin) and a flavanone (Naringin) were identified (Fig. 1). The detection of compounds was achieved by using Agilent ChemStation software (Agilent technology, USA). Authentic compounds were used to identify and quantify flavonoids and phenolics in CR extract. This study is the first to report the polyphenol composition of CR leaf extract.

The histopathological and immunohistochemical studies proved that CR extract greatly protects the liver tissue from the steato-cirrhotic effect of CCl_4_. The exposure of the rats to CCl_4_ causes hepatocytes vacuolation, and induces hepatic inflammation and fibrosis as previously reported [47]. The CR extract significantly decreased the collagen deposition, and suppressed the inflammatory cellular infiltrations. Tumor necrosis factor α (TNF α) is a one of cytokines produced mainly by macrophages, that stimulates cellular response such as production of the inflammatory mediators and cell death [48]. In current study, the CCl_4_ treated group showed a significant increase in TNF α in comparison to CR treated group. This was attributed to the role of CR extract as an anti-inflammatory agent. Arginine is a substrate for the synthesis of urea and nitric oxide, arginase has a catalytic activity which converts arginine to urea [40]. In the present work, the arginase showed a significant decrease in CR treated group in comparison to CCl_4_, and its elevation in the CCl_4_, intoxicated group occurred as a result of the up regulation of TNF α [49].

The accumulation of reactive oxygen species (ROS) within the cells along with a decrease in the cellular antioxidants provokes mitochondrial damage [50]. This led to the release of the apoptogenic factors (Cytochrome c) through the mitochondrial membrane. Bcl-2 is an anti-apoptotic member which inhibits or decreases apoptosis [51]. The CR extract exhibited a significantly increase in Bcl-2 positive hepatocytes which indicates its role in the protection of the hepatocytes from the programed cell death. Additionally, our results showed a significant increase in PPAR α in the CCl_4_ intoxicated group due to the elevation of intercellular lipid accumulation. PPAR α mainly controls the lipid metabolism and regulates the inflammatory response in liver [41]. In this regard, the increase in the number of PPAR α positive cells is due to the excess of the inflammatory cell’s infiltration after the exposure to CCl_4_ [52]. PPARα levels decreased in the CR extract group [53]. Thus, CR ethyl acetate extract has a prospective anti-steatosis activity.

Molecular docking is a powerful approach that has been employed in drug discovery studies to screen the binding affinities of the drugs with its possible target proteins [54]. Therefore, we used this approach to study the inhibitory role of the identified polyphenols on Bcl2: Bim (BH3) as a crucial pro-apoptotic protein. Interestingly, naringin, rutin, hydroxy flavone, and ellagic acid showed a promising binding affinity with Bcl2: Bim (BH3). These high binding affinities are attributed to the unique structure of polyphenols that combine the aromatic rings and the substituted polar groups that allow them to form various non-covalent interactions with the target proteins. These findings suggested the antiapoptotic role of polyphenols through the inhibition of BH3 protein.

## Conclusion

The ethyl acetate extract of *C. rotang* L. displayed significant in vivo hepatoprotective effect on CCl_4_ induced hepatotoxic rats. The current study confirms its potent antioxidant, anti-inflammatory and anti-steatosis activities through the suppression of TNF α, arginase and PPAR α induced by CCl_4_. The antiapoptotic property of the extract was through the enhancement of Bcl-2 antiapoptotic protein. DPPH antioxidant activity, total phenolic contents, total flavonoid contents and HPLC analysis revealed that *C. rotang* L. ethyl acetate extract is a rich source of antioxidant and anti-inflammatory metabolites. Fourteen phenolic and flavonoid compounds were detected. Their docking on proapoptotic protein Bcl-2 (BH3) led to the detection of naringin, rutin, 7-hydroxy flavone and ellagic acid as the highest affinity compounds to BH3.

## Supplementary Information

**Additional file 1. **

## Data Availability

Correspondence and requests for materials should be addressed to A.M.Z. and F.M.A.

## Abbreviations

HPLC: High Performance Liquid Chromatography
Bcl-2: Beta cell lymphoma 2
TNFα: Tumer Necrosis Factor Alpha
DPPH: 2,2-Diphenyl-1-picrylhydrazyl
PPARα: Peroxisome Proliferator-Activated Receptor Alpha
°C: Celsius temperature degree
mg/kg: Milligram/kilogram
nm: nanometer
UV/Vis: Ultraviolet/Visible
GAE: Gallic acid Equivalent
QE: Quercetin Equivalent
SD: Standard Deviation
CR: *Calamus rotang*
µM: Micromolar
µl: Microliter
ppm: Part per million
g: Gram
CCl_4_: Carbon Tetra Chloride
Bcl-2:BH3: Beta cell lymphoma 2 Homology 3
PDB ID: 4B4S: Crystal structure of a pro-survival Bcl-2:Bim BH3 complex
