# Correction: Potent hepatoprotective activity of common rattan (*Calamus rotang* L.) leaf extract and its molecular mechanism

**DOI:** 10.1186/s12906-023-03881-5

**Published:** 2023-02-20

**Authors:** Walaa S. Anwar, Fatma M. Abdel-maksoud, Ahmed M. Sayed, Iman A. M. Abdel-Rahman, Makboul A. Makboul, Ahmed M. Zaher

**Affiliations:** 1 Department of Pharmacognosy, Faculty of Pharmacy, Assiut University, Assiut, 71515 Egypt; 2 Department of Anatomy & Embryology, Faculty of Vet. Medicine, Assiut University, Assiut, Egypt; 3 Biochemistry Laboratory, Chemistry Department, Faculty of Science, Assiut University, Assiut, Egypt; 4 Department of Pharmacognosy, Faculty of Pharmacy, South Valley University, Qena, Egypt; 5 Department of Pharmacognosy, Faculty of Pharmacy, Merit University, New Sohag, Egypt

**Correction: BMC Complement Med Ther 23, 24 (2023)**

**https://doi.org/10.1186/s12906-023-03853-9**

Following the publication of the original article [1], it was noted that due to a typesetting error Dr. Walaa S. Anwar and Dr. Makboul A. Makboul were incorrectly affiliated to Merit University.

Correct affiliation for Dr. Walaa S. Anwar and Dr. Makboul A. Makboul should be Department of Pharmacognosy, Faculty of Pharmacy, Assiut University, 71515 Assiut, Egypt.

The affiliations have been updated above and the original article [1] has been corrected.

## References

[1] Anwar WS, Abdel-maksoud FM, Sayed AM (2023). Potent hepatoprotective activity of common rattan (Calamus rotang L.) leaf extract and its molecular mechanism. BMC Complement Med Ther.

