# Supplementary material for: Potent hepatoprotective activity of common rattan (Calamus rotang L.) leaf extract and its molecular mechanism
Source: BMC Complement Med Ther. 2023 Jan 30;23:24. doi: 10.1186/s12906-023-03853-9 (PMC9885597; doi:10.1186/s12906-023-03853-9)
Supplement: Supplementary file 1 — Additional file 1. [file 12906_2023_3853_MOESM1_ESM.docx]

**Potent hepatoprotective activity of common rattan (*Calamus rotang* L.) leaf extract and its molecular mechanism**

**Walaa S. Anwar^1^, Fatma M. Abdel-maksoud^2^,** **Ahmed M. Sayed^3^,** **Iman A. M. Abdel-Rahman^4^,** **Makboul A. Makboul^1^, Ahmed M. Zaher^1*^**

^1^ Department of Pharmacognosy, Faculty of Pharmacy, Assiut University, 71515

Assiut, Egypt.

^2^ Department of Anatomy, Faculty of Vet. Medicine, Assiut University, Assiut, Egypt.

^3^ Biochemistry Laboratory, Chemistry Department, Faculty of Science, Assiut University, Assiut, Egypt.

^4^ Department of Pharmacognosy, Faculty of Pharmacy, South Valley University, Qena, Egypt.

**
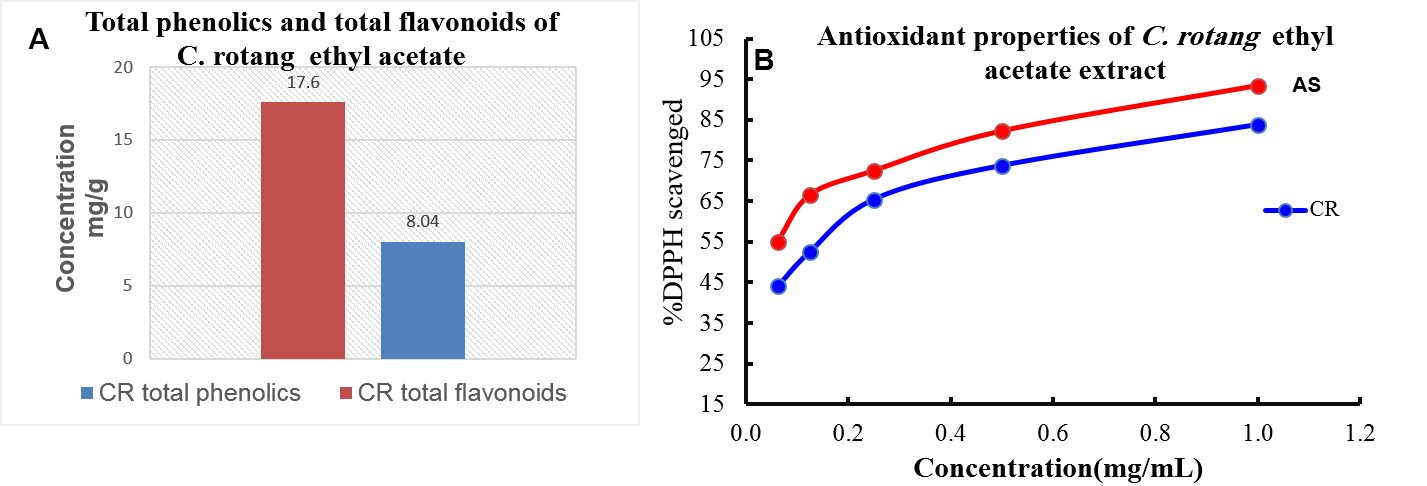
**

**Fig.S1: A) Phenolics and flavonoids contents of *C. rotang* L. ethyl acetate extract determined by Folin-Ciocalteu and Aluminum chloride colorimetric methods.**

**B) DPPH scavenged (%) activity (SA%) of *C. rotang* L. (CR) and ascorbic acid (AS). (**SA% = (A_0_ - A_s_/A_0_) × 100) (A_0_ = Absorbance of DPPH solution in ethanol, A_s_ = Absorbance of CR extract or ascorbic acid and DPPH solution).

**
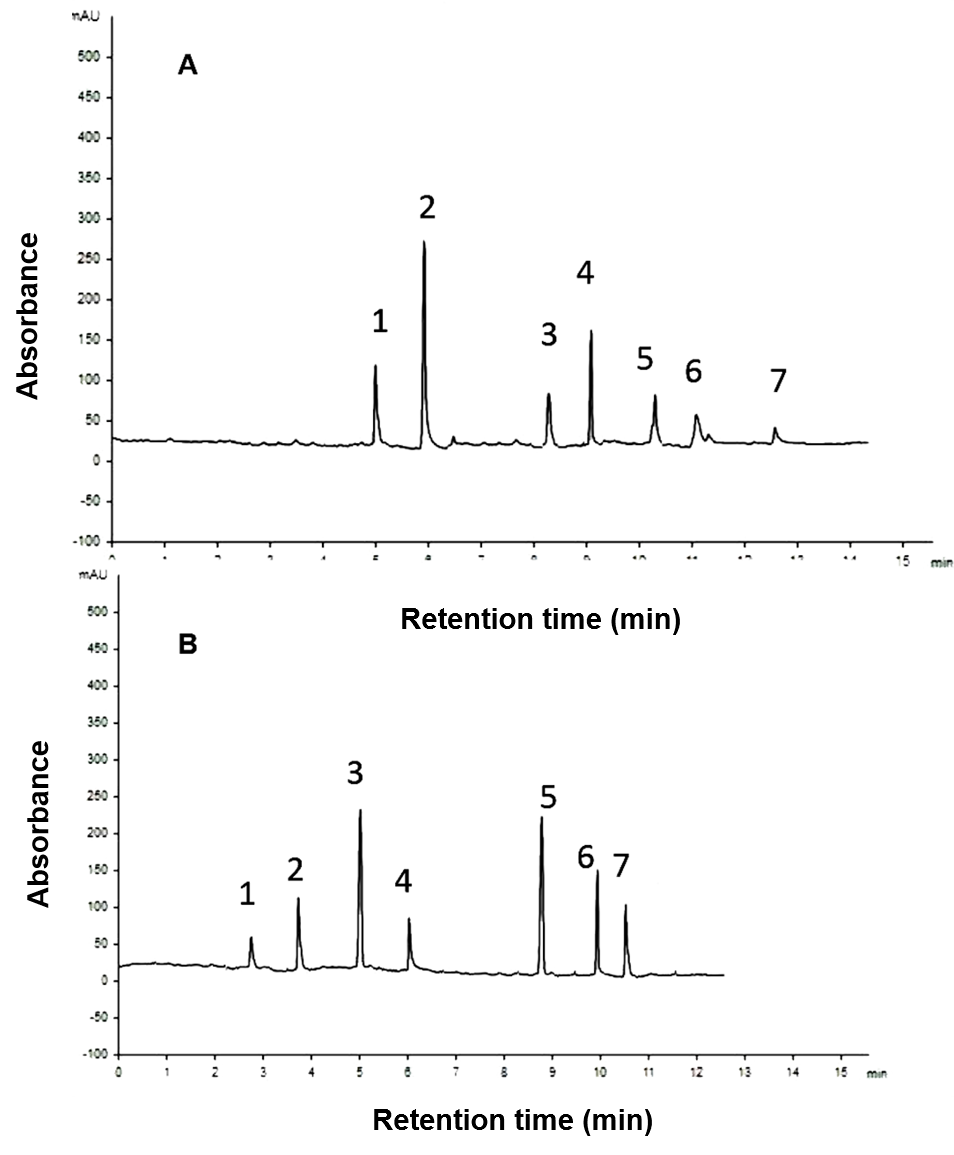
**

**Fig.S2: A) HPLC chromatogram of detected phenolics of *C. rotang* L. ethyl acetate. Seven phenolics were identified using authentic samples by matching the retention time (min).**

**B) HPLC chromatogram of detected flavonoids of *C. rotang* L. ethyl acetate extract. Seven flavonoids were identified using authentic samples by matching the retention time (min).**

**Fig S3:** **Standard curve of gallic acid used in Folin-Ciocalteu assay for detection of phenolic contents.**

**Fig. S4.:** **Standard curve of quercetin used in Aluminum chloride assay for detection of flavonoids content.**

**
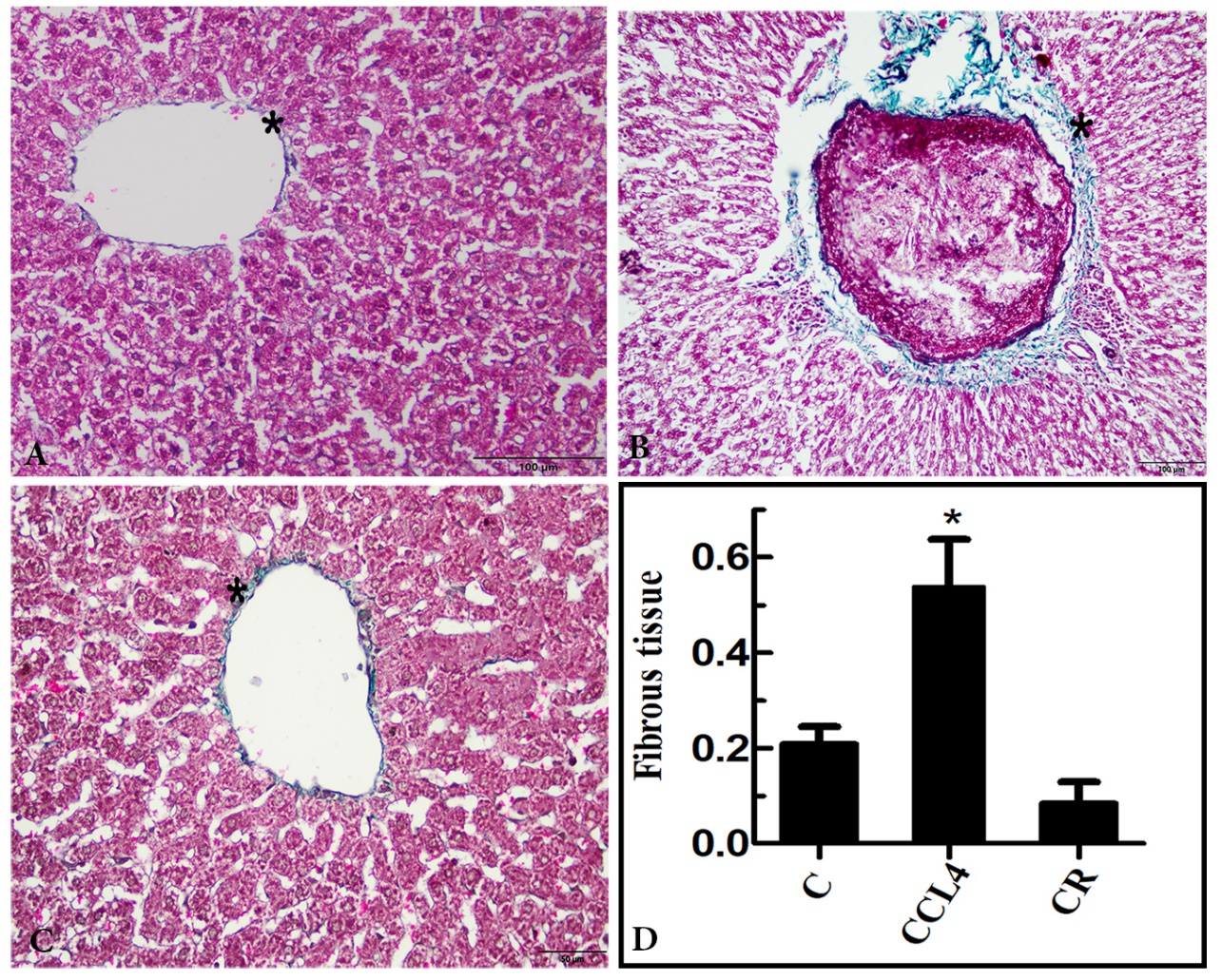
**

**Fig. S5: Photomicrograph of a liver sections stained with Crossman’s trichrome.** A: The mild green staining for collagen around the central vein (C) and portal tract (P) in normal group, B: the strong staining for collagen fibers in CCL_4_ group. C: The deposition of collagen in CR extract group was lesser. D: Quantification of Crossman’s trichrome staining using Image-J software. The CR extract treated group is compared to control and CCL_4_ groups using Tukey test (n = 3). *P ≤ 0.05.

**Table S1**: Molecular docking of identified metabolites as potential inhibitors of pro-apoptotic Bcl2: Bim (BH3) protein

| **Compounds** | **Lowest energy of docking (kcal/mol)** |
| --- | --- |
| Myricetin | -6.10±0.41 |
| Quercetin | -6.23±0.30 |
| Apigenin | -6.35±0.33 |
| Kaempferol | -6.05±0.34 |
| Naringin | -6.88±0.06 |
| Rutin | -6.70±0.19 |
| 7-hydroxy flavone | -6.42±0.31 |
| Ellagic acid | -6.57±0.26 |
| Syringic acid | -4.48±0.09 |
| Gallic acid | -4.82±0.19 |
| Caffeic acid | -4.98±0.24 |
| Ferulic acid | -4.75±0.15 |
| *P*-coumaric acid | -4.58±0.16 |
| Pyrogallol | -4.47±0.13 |
